# Microbiology laboratories involved in disease and antimicrobial resistance surveillance: Strengths and challenges of the central African states

**DOI:** 10.4102/ajlm.v11i1.1570

**Published:** 2022-03-31

**Authors:** Passoret Vounba, Severin Loul, Ludovic F. Tamadea, Joël F.D. Siawaya

**Affiliations:** 1 Economic Community of Central African States (ECCAS) Commission/Fourth phase of the Regional Disease Surveillance Systems Enhancement Project (REDISSE IV), Libreville, Gabon; 2 Department of Laboratory Services, CHU Mère-Enfant Fondation Jeanne EBORI, Libreville, Gabon; 3 Regional Integrated Surveillance and Laboratory Network (RISLNET) for Central Africa, Libreville, Gabon

**Keywords:** laboratory capacity, Economic Community of Central African States (ECCAS), laboratory strengthening, One Health, epidemics, antimicrobial resistance

## Abstract

Laboratory systems have been largely neglected on the margins of health systems in Africa. However, since the 2000s, many African countries have benefited from massive investments to strengthen laboratory capacities through projects fighting priority diseases (HIV/AIDS, tuberculosis, malaria). This review examined the laboratory capacities of the Economic Community of Central African States (ECCAS). Online research using specific terms was carried out. Studies published between 2000 and 2021 on the role of the laboratory in disease and antimicrobial resistance surveillance in the 11 ECCAS countries were considered. The number of human and animal health laboratories meeting international standards was very low in the sub-region. There were only seven International Organization for Standardization (ISO) 15189-accredited human health laboratories, with five in Cameroon and two in Rwanda. There were five high biosafety level (BSL) laboratories (one BSL3 laboratory each in Cameroon, the Central African Republic, Democratic Republic of Congo and the Republic of Congo, and one BSL4 laboratory in Gabon) and three ISO 17025-accredited laboratories in the ECCAS sub-region. Only six countries currently have whole-genome sequencing devices, which is insufficient for a sub-region as large and populous as ECCAS. Yet, a plethora of pathogens, particularly haemorrhagic viruses, are endemic in these countries. The need for laboratory capacity strengthening following a One Health approach is imperative. Since emerging and re-emerging zoonotic infectious diseases are projected to triple in frequency over the next 50 years and given the inextricable link between human and animal health, actors in the two health sectors must collaborate to preserve world health.

## Introduction

Laboratory results guide evidence-based clinical decisions. In 1923, Louis Pasteur declared that ‘without laboratories, men of science are soldiers without arms’.^1^ A standard medical laboratory is essential for patient care and communicable disease surveillance. The laboratory is indispensable, both for routine diagnosis of infections and for the rapid identification of epidemic outbreaks. In addition, antimicrobial resistance (AMR) surveillance, food safety and water quality assessment, analysis of environmental samples, etc. are also laboratory dependent. In sub-Saharan Africa, unfortunately, laboratory service has been a sidelined health service that receives very little government budgetary allocation. In 2008, representatives of African governments recognised that:

In resource-limited settings, several challenges have resulted in inadequate laboratory systems to support the scale-up of programs. These include a lack of leadership and advocacy, human resources, career path and retention of staff, national laboratory policy, strategic planning (budgetary concerns), sufficient physical infrastructure, supply chain management, and quality management systems (quality assurance). (p. 1)^2^

The limited investment in laboratory systems impairs the quality of laboratory services. For instance, sub-Saharan African laboratories are deficient in terms of qualified staff, modern equipment, a regular supply of quality reagents, water, or electricity, standard operating procedures, and quality assurance systems.^3,4^ This review aimed to highlight the roles, strengths, and challenges of the human and animal laboratories in the Economic Community of Central African States (ECCAS).

The ECCAS is an integrated African space created in 1983 and includes 11 countries: Angola, Burundi, Cameroon, Central African Republic, Chad, Democratic Republic of Congo, Equatorial Guinea, Gabon, Republic of Congo, Rwanda, and São Tomé and Príncipe. According to the 2014 estimates, the population of the ECCAS is about 161 million inhabitants, spread over an area of 6 640 490 km^2^.^5^

A systematic search was carried out using terms ‘role of the laboratory in Africa’, ‘role of laboratory in disease surveillance in Africa’, ‘role of veterinary laboratories in Africa’, ‘role of the laboratory in AMR surveillance in Africa’, ‘ISO [International Standardization Organization] 15189-accredited laboratory in Africa’, ‘ISO 17025-accredited laboratory in Africa’, ‘health reference laboratories in Africa’, ‘veterinary reference laboratories in Africa’, and each term accompanied by the name of each of the 11 ECCAS countries. These terms were searched on Google, Google Scholar, PubMed, African Journals Online and international organisations’ websites: (World Health Organization [WHO], World Organization for Animal Health [OIE], Food and Agriculture Organisation of the United Nations, Africa Centres for Disease Control and Prevention, African Society for Laboratory Medicine [ASLM]). All studies published between 2000 and 2021 in the four official languages used by the ECCAS (English, French, Spanish and Portuguese) on the role of the laboratory in the surveillance of diseases and AMR in the 11 countries were included.

## Results and discussion

### Role of human health laboratory in patient management

Medical laboratories provide a precise diagnosis for patient management. Early diagnosis and treatment reduce the risk of long-term complications in patients and prevent further transmission.^6^ Thus, proper patient management requires close tripartite collaboration between the patient, clinicians and laboratory staff.^7^

In sub-Saharan Africa, access to reliable diagnostic tests is very limited and misdiagnosis occurs frequently, leading to physicians’ distrust of laboratory results.^6^ Limited access to reliable diagnosis results in no or inadequate treatment, increased mortality, and an inability to determine the true prevalence of diseases.^8^ In the Democratic Republic of Congo, the laboratories were unable to diagnose the diseases frequently encountered in this country.^4^ The lack of laboratory infrastructure may delay patients’ recovery. Delay may occur due to referrals to other laboratories that are further away and often more expensive, or due to inappropriate treatment that lacks laboratory diagnosis. Arsuaga et al.^9^ reported an example of the latter: the case of a missionary symptomatically diagnosed and unsuccessfully treated for malaria in Cameroon and Equatorial Guinea, but was laboratory diagnosed and successfully treated for babesiosis, a malaria-like illness, eight months later in Spain.

In Rwanda, a strong laboratory network was built in the early 2000s. Internal and external laboratory control and assurance activities are regularly conducted at all levels of the network by the National Reference Laboratory (NRL).^10^ The external quality assessment focuses on enteric and meningitis pathogens, tuberculosis, malaria, and HIV/AIDS. In 2003, a concordance of 100% was reported for the unlinked, anonymous HIV/AIDS testing of all 288 samples sent by the Rwandan NRL to the United States Centre for Disease Control and Prevention.^10^ Nevertheless, Rwandan laboratories are not exempt from cross-cutting problems, such as service interruptions due to reagent stock-out and equipment breakdown.^11^ The positive impact of laboratory capacity building on reducing infections and improving patient management has been reported in Cameroon. Eleven months after implementing capacity strengthening activities, the regional hospital of Buea reported a reduced patient wait time at the reception from 3 h to less than 30 min.^12^ Similarly, laboratory improvement capacities in the Bamenda Regional Hospital Laboratory resulted in fewer specimen recalls, improved test reliability, and the provision of feedback channels on services offered.^13^

From the ECCAS sub-region, Gabon, Cameroon, the Democratic Republic of Congo, and the Central African Republic are part of the WHO’s Emerging Dangerous Pathogens Laboratory Network.^14^ All four countries have national Viral Haemorrhagic Fever (VHF) testing capacity, while Gabon hosts the WHO AFRO ECCAS regional VHF reference laboratory.^15^ All ECCAS countries have influenza laboratories except Chad, Equatorial Guinea, and São Tomé and Príncipe. However, the influenza laboratory network in the Republic of Congo, Angola, and Rwanda can easily be upgraded to include VHF testing capacities.^15^

In Cameroon, the *Centre Pasteur du Cameroun* is a reference centre for the network of quantitative polymerase chain reaction diagnostic laboratories for Buruli ulcer. This network brings together 11 laboratories located in nine West and central African countries where Buruli ulcer is endemic.^16^ Ultimately, other neglected tropical diseases such as leprosy and cutaneous leishmaniasis will be integrated into the Buruli ulcer platform. The network also plans to implement activities such as clinical trials evaluating new treatments, assays validating new molecular diagnostic tools, and surveillance of AMR.

### Role of laboratories in disease surveillance in the ECCAS sub-region

The information provided by the laboratory is critically important for disease surveillance and response programmes. For efficient management of an epidemic and its containment, a strong laboratory system should be operational before, during, and after the epidemic.^17,18^ Before an epidemic, the laboratory collects early warning signals and identifies the aetiological agent. During the outbreak, the laboratory is involved in the response and management measures for the containment of the epidemic, and after the outbreak, the laboratory monitors disease trends, evaluates interventions, and monitors progress towards control objectives.

Apart from disease outbreaks, laboratories monitor microbial genetic changes of public health concerns, such as changes that confer AMR in bacteria or changes in RNA viruses (such as Ebola or coronavirus) that lead to the emergence of genetically diverse strains (variants) with high pathogenicity or transmissibility.^19^ Thus, during certain outbreaks, such as the Ebola or coronavirus outbreaks, the aetiological agent must be laboratory-characterised to detect the emergence of variants and guide response decisions.^18^

Detection of extremely dangerous pathogens, such as the Ebola virus, requires higher biosafety level (BSL3 or 4) laboratories. BSL4 laboratories are built to ensure biosafety and biosecurity when studying class 4 pathogens; pathogens transmitted via aerosols or unknown mechanisms and are often lethal, without known treatment or vaccine to fight them. However, in the absence of a BSL4 laboratory such as in the Democratic Republic of Congo, BSL3 laboratories, for studying class 3 pathogens, usually, viruses or bacteria that infect humans or animals through inhalation and could be lethal, with reinforced biosafety and biosecurity, have been used to diagnose Ebola cases.

Africa experiences approximately 100 public health events every year, of which 80% are caused by infectious agents.^14^ Many of these events involve extremely dangerous pathogens. For example, since 1994, the ECCAS region has regularly recorded Ebola epidemics, mainly in the Democratic Republic of Congo, the Republic of Congo, and Gabon,^20^ which due to their ecosystems are at high risk of VHF.^21^

Unfortunately, despite the high health risks evident in African countries, resources for epidemic surveillance and response such as laboratory capacity are mostly lacking. As of 2015, across the entire African continent, only three countries (Nigeria, Kenya and South Africa) had fixed BSL3 laboratories, while only two countries had BSL4 laboratories^22^: one in Gabon, *Centre Interdisciplinaire de Recherches Médicales de Franceville (CIRMF)*, and the other in South Africa. As at March 2021, the number of operational BSL4 laboratories on the continent has not much changed: two are under construction in South Africa and Côte d’Ivoire^23^; the number of BSL3 laboratories has increased, particularly in the ECCAS sub-region.

BSL3 laboratories were recently built, including the *Institut National de Recherches Biomédicales* in the Democratic Republic of Congo, the *Centre Pasteur du Cameroun* in Cameroon, the *Institut Pasteur de Bangui* in the Central African Republic,^14^ and the BSL3 laboratory dedicated to the management of multidrug-resistant tuberculosis in the Republic of Congo.^24^

The presence of the BSL3 and BSL4 laboratories in some ECCAS countries is a great asset for the sub-region to effectively monitor and respond to epidemics. For instance, the BSL4 laboratory in the CIRMF, Gabon, actively surveils emerging and re-emerging diseases not only in Gabon but also in the other ECCAS countries.^25^ The objectives assigned to the CIRMF includes diagnosing suspected VHF cases, developing new diagnostic methods, monitoring deaths in animal reservoir hosts, and conducting laboratory techniques training at national, regional and international levels. The CIRMF has established a research partnership with the National Public Health Laboratory in Brazzaville, Republic of Congo, and the *Institut National de Recherches Biomédicales* in Kinshasa, Democratic Republic of Congo, to study infectious diseases transmitted by animals in the tropical rainforest regions of equatorial Africa.^25^

#### Capacities of the human health laboratories in ECCAS sub-region to comply with international health regulations

In 2015, the WHO recommended that member states annually report their progress in implementing the revised international health regulations (2005 IHR)^26^ and conduct a self-assessment of their capacity, followed by a joint external evaluation (JEE).^26^ The JEE tool is developed using the WHO instruments as well as different strategies and initiatives including the Global Health Security Action Programme and the OIE tool for the evaluation of the performance of veterinary services (PVS).^26^ The JEE assesses IHR capacities in 19 technical areas, grouped into four main themes: ‘prevention’, ‘detection’, ‘response’, and ‘entry points and other IHR risks’ (chemicals and radiation).

**FIGURE 1:**
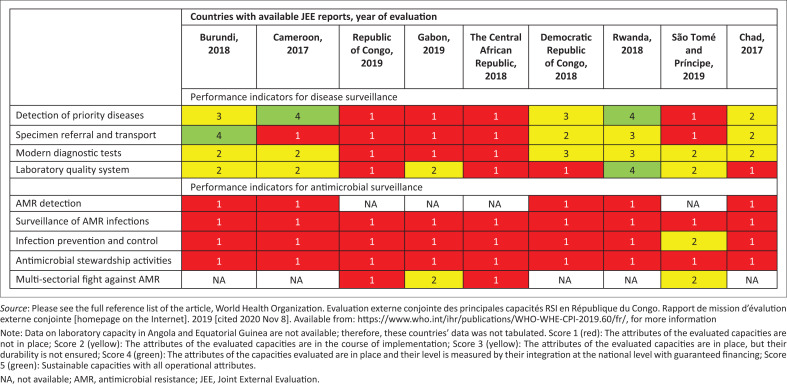
Country scores for laboratory capacity assessment in the ECCAS countries.

A national laboratory system is one of the four technical areas of the domain ‘detection’. The 2005 IHR capacity scores are classified from level 1 (no capacity) to level 5 (sustainable capacity). In the ECCAS region, except Angola and Equatorial Guinea, all countries have completed the JEE of their public health capacity to meet the requirements of the 2005 IHR. As per Figure 1, in the laboratory area, most ECCAS countries scored low in several indicators both in terms of disease and AMR surveillance capacities.^24,27,28,29,30,31,32,33,34^

#### Capacities of the animal health and food laboratories in the ECCAS sub-region to comply with the international standards

In animal health and food safety, the performance evaluation of the veterinary services has shown that laboratory reliability and quality assurance are major issues in most African countries.^35^ For ECCAS countries, evaluation or gap reports reveal that laboratory capacities are very low for disease and AMR surveillance. These weaknesses are both qualitative (very low performance scores, around 1–2 for the majority of countries) and quantitative (often only one regional or national veterinary laboratory per country, rarely two) (Supplementary Table 1).

**TABLE 1:** Role of the laboratory system in achieving the goals of the WHO’s global plan against AMR.

WHO’s strategic objectives	In clinical care	In public health
1) Increase awareness and understanding of AMR through communication, education and training	Adequate training of clinicians and other healthcare professionals on AMR. Patient education to reduce unnecessary demand for antimicrobials.	Updated AMR reports to ministries of health and policymakers. Inform the media and all stakeholders about AMR. Communicate the threat of irrational antimicrobial use.
2) Strengthen knowledge and evidence base through surveillance and research	Correctly identify the aetiology of human and animal infections. Monitor the effectiveness of antibacterial treatment. Communicate the results of antibiograms. Pilot and implement new technologies that could increase the access and speed of testing or reduce its cost.	Accelerate the search for rapid diagnosis of infections. Implement quality assurance for antibiotic susceptibility testing. Develop AMR surveillance strategies at the human-animal-ecosystem interface. Develop surveillance plans based on national AMR laboratories. Implement antimicrobial stewardship programmes.
3) Reduce the incidence of infections through effective sanitation, hygiene and infection control measures	Support infection control by identifying and separating patients infected with resistant pathogens. Allow tracking of sources for infections.	Promote rapid and effective antibiotic therapy so that pathogens are less likely to be transmitted. Prevent epidemics through early identification of outbreaks and improved management and containment. Provide laboratory support to assess health risks.
4) Optimise the use of antimicrobial drugs in human and animal health	Replace the broad-spectrum regimen with narrow-spectrum drugs, thereby reducing the risk of antibiotic-associated infections.	Promote the application of surveillance data to national and regional pharmaceutical policy. Preventive withdrawal of certain antimicrobials in livestock, especially those that could cause cross-resistance with antimicrobials used in human health.
5) Make the economic case for sustainable investments	Reduce drug costs by allowing the cheapest effective drug to be selected rationally.	Determine the true cost of AMR to provide economic evidence to support the replacement of antimicrobial use with vaccines and other preventive strategies.

*Source*: Please see the full reference list of the article, World Health Organization. Plan d’action mondial pour combattre la résistance aux antimicrobiens [homepage on the Internet]. Genève: OMS, 2015 [cited 2020 Dec 19]; 32 p. Available from: https://www.who.int/antimicrobial-resistance/global-action-plan/fr/, for more information

AMR, antimicrobial resistance; WHO, World Health Organization.

To achieve the goal of eliminating dog-mediated human rabies deaths by 2030, many African veterinary laboratories, including the *Laboratoire National Vétérinaire* (LANAVET) in Cameroon and the Veterinary Laboratory of Kinshasa, Democratic Republic of Congo, recently benefited from increased capabilities for rabies diagnosis. The staff were trained to diagnose rabies using the direct fluorescence antibody test and conventional real-time polymerase chain reaction at the Food and Agriculture Organisation of the United Nations Rabies Reference Centre in Italy.^36^

### Mobile laboratories as a solution to the lack of fixed laboratory infrastructures

In sub-Saharan Africa, particularly ECCAS countries, the few BSL3 and BSL4 laboratories are located in large urban centres. Thus, they could be located far from epidemic outbreaks or areas at risk of emergence or re-emergence of epidemics. In the case of an epidemic such as Ebola, the safe delivery of samples to the laboratory, reliable diagnosis, and prompt communication of results are crucial for a successful response.^37^ A mobile laboratory can alleviate this problem by shortening the time required to obtain results. Mobile laboratories circumvent fixed laboratory construction delays, particularly in times of emergencies, as they can be deployed almost immediately. This has been demonstrated in some countries in the sub-region.

During the 2005 Ebola epidemics in the Democratic Republic of Congo and the 2007 Marburg fever epidemic in Angola, mobile laboratories confirmed suspected cases within 4 h, consequently facilitating the work of the contact-tracing team.^38^ More recently, a portable sequencer in one of the mobile laboratories was used to investigate the date of introduction and geographical origin of the Zika virus in Angola.^39^ However, field mobile laboratories are capital- and logistics-intensive, and are thus best suited for providing limited services for brief periods.^18^ Therefore, it is necessary to develop additional fixed and sustainable BSL3 and BSL4 laboratories in the ECCAS.

### Role of laboratories in AMR surveillance

#### Overview of the challenges of AMR surveillance

The advent of antibiotic therapy, which began with the discovery by 1928 of penicillin, completely revolutionised medicine and significantly reduced infectious disease mortality and disability. The use of antimicrobials has also increased animal production by improving animal welfare. Unfortunately, AMR seriously undermines the hopes raised by the discovery of antimicrobials. Antimicrobial resistance is considered one of the most significant threats to human, animal and ecosystems health. Antimicrobial resistance is exacerbated by the overuse and misuse of antimicrobials; 30% – 50% of antimicrobial prescriptions in human medicine are unnecessary.^40,41,42^

In animal health, the irrational use of antimicrobials is compounded by the use of growth promoters in animals or the use of antimicrobials for metaphylaxis. Some growth promoters contain antimicrobials of critical importance to human health.^43^ In addition, in veterinary medicine metaphylaxis is rampant; metaphylaxis is the administration of antimicrobials to a herd of animals to treat sick individuals and prevent the disease in healthy individuals.

Antimicrobial resistant microorganisms in animals can subsequently be transmitted to humans through direct or indirect contact.^44^ Indeed, there is a much higher risk of human colonisation through cattle, pigs and poultry infected with methicillin-resistant *Staphylococcus aureus*.^45^ Furthermore, genetic determinants of AMR can be transferred from commensal or pathogenic animal bacteria to pathogenic human bacteria. Last resort antimicrobials used to fight multidrug-resistant infections such as fluoroquinolones, third generation cephalosporins or colistin are becoming ineffective globally.

In parallel to the extensive misuse of antimicrobials, the discovery of new antimicrobials has become increasingly seldom. As a result, the feared therapeutic impasse is becoming increasingly real.^46^ According to projections, by the year 2050, AMR will be the world’s leading cause of annual death, with 10 million deaths per year, ahead of cancers (8.2 million) or diabetes (1.5 million).^47^ Also, Africa and Asia will likely be the most affected continents. This is why, on the margins of the 71st session of the United Nations General Assembly in 2016, the alarm bell was sounded on AMR.^48^ On this occasion and for the first time, heads of states and governments came together to adopt a common approach to fight the causes of AMR in human and animal health, as well as in the environment. In this noble fight, the laboratory has a prominent part to play.

#### Role of the laboratory in fighting AMR

The early symptoms of an infectious disease may be non-specific and may combine clinical signs of several infectious diseases. For example, the first manifestations of an Ebola virus infection include fever, headache, myalgia, and gastrointestinal disorders.^49^ To increase the chance of effective antibiotic therapy, a common approach is to use broad-spectrum antimicrobials while waiting for antimicrobial susceptibility test results, which are not usually available before 72 h.^50^ This practice runs contrary to the goal of the WHO global plan to optimise antimicrobial use.^51^ This empirical usage selects for antimicrobial-resistant microorganisms. Therefore, medication before laboratory diagnosis should only be used when the disease is life-threatening and, even so, microbiological sampling should be performed before treatment is initiated.^52^ The focus should be on the development of innovative rapid diagnostic techniques that allow clinicians to identify the pathogen in minutes rather than days.^53^

Antibiotic susceptibility testing is a key indicator for the design of effective interventions and rational use of antibiotics.^54,55^ Reporting of antibiotic susceptibility results by medical laboratories is necessary to monitor emerging resistances and develop appropriate antimicrobial stewardship guidelines.^54,56^ Therefore, antimicrobial susceptibility testing capacity is essential.^57^

In the 2018–2023 AMR framework,^58^ the Africa Centre for Disease Control and Prevention, in collaboration with existing partners, aimed to increase laboratory capacity for the detection of resistant microorganisms in humans and animals. The WHO’s global plan of action defines five strategic objectives for AMR containment.^45^ As per Table 1, the laboratory system has a key role to play in achieving these goals, both in terms of clinical and public health activities.^45,51,59,60,61,62^

In the ECCAS countries, probably due to weak laboratory capabilities, there is a huge AMR data gap from the human,^63^ animal and environmental health sectors.^64^ However, the literature suggests that substantial effort is being made to achieve some of the objectives of the WHO’s global plan against AMR. Scientific articles contribute to public awareness, understanding, and knowledge building on AMR. In Belgium, for example, as a result of national awareness campaigns, *Streptococcus pneumoniae* penicillin resistance decreased from 18% in 2000 to 7% in 2009.^65^ Thus, in the ECCAS zone, research and public awareness should be at the heart of global AMR control strategies.

In animal health, the LANAVET in Cameroon and the *Institut de Recherche en Elevage pour le Développement* in Chad are major vaccine production laboratories in Africa^66^ aimed at preventing infections, thereby reducing antimicrobial use in animals.

### Next-generation sequencing capacity in the ECCAS region

Next-generation sequencing (NGS) offers the potential to provide more accurate and timely information, thereby increasing the likelihood of meeting the 2005 IHR recommendations. The IHR recommends that urgent events are reported within 48 h to determine whether an event is ‘notifiable’. This information will rapidly inform the necessary control measures to prevent national and international transmission. Diagnosis and surveillance of pathogens are the core capacity of public health systems.^67^ The whole-genome sequencing is a leading technique in the response, not only to the ongoing coronavirus disease 2019 pandemic but to future emerging and re-emerging infections.

In the ECCAS region, countries with NGS devices are Gabon (four devices), the Democratic Republic of Congo and Rwanda (two devices each), Angola and Cameroon (one each),^68^ and the Republic of Congo and Equatorial Guinea (unknown number each).^69^ The Illumina platform is by far the most used in these countries, followed by Ion Torrent and Nanopore.

### ISO 15189- or ISO 17025-accredited laboratories in the ECCAS region

The WHO AFRO through the African Society of Laboratory Medicine (ASLM), implemented the Stepwise Laboratory Management Towards Accreditation (SLMTA), to improve medical laboratories in Africa.^70^ The SLMTA was launched in 2009 to improve the quality of public and private health laboratories in African countries to achieve ISO 15189 standards accreditation. The framework to audit the implementation of the SLMTA in laboratories is the Stepwise Laboratory Improvement Process Towards Accreditation (SLIPTA).^70^ Until October 2021, there were only two ECCAS countries with ISO 15189-accredited laboratories: Cameroon had five accredited laboratories while Rwanda had two.^71^

Although Cameroon scored very low on most capacity attributes in the JEE (Figure 1), it has the largest number of accredited laboratories in the ECCAS region. The likely explanation could be that these currently accredited laboratories were not included in the JEE cohort or that they rigorously accelerated their certification process after the JEE. The last decade has seen the emergence of several projects supporting laboratory systems in low-income countries, notably as part of the fight against priority diseases (malaria, HIV/AIDS and tuberculosis). These projects have positively impacted laboratory services.

The accreditation of veterinary laboratories is subject to ISO 17025 standards. By the end of the European Union-funded Central African Quality Infrastructure Project (PIQAC) in 2019, two food safety laboratories had been audited and were in the process of ISO 17025 accreditation.^72^ Six others were in the process of capacity building for accreditation, including the microbiology laboratory in the *Centre de Contrôle de la Qualité des Denrées Alimentaires* (CECOQDA) in N’Djamena, Chad.^72^ As of March 2021, the CECOQDA’s microbiology laboratory with the Congolese Office Control Laboratory in the Democratic Republic of Congo and the Africa Improved Food laboratory in Rwanda were the few ISO 17025-accredited laboratories in the ECCAS region. In addition, the LANAVET in Cameroon is considered a centre of laboratory excellence by the Food and Agriculture Organisation of the United Nations.^73^ This laboratory organises training sessions on animal disease diagnosis for technicians in the sub-region and provides African swine fever diagnostic services for Chad.

### Capacity building needs of laboratories in the ECCAS zone

The ECCAS countries need to incite both medical and veterinary laboratories to register in ISO 15189 or ISO 17025 accreditation processes. Accreditation assessments are snapshot measurements of laboratory compliance, creating the risk that the efforts may weaken after an assessment.^74^ Therefore, for already accredited laboratories, the most important challenge is maintaining their status.

According to 2017 forecasts, the world’s population is expected to increase by 2.2 billion by 2050, with 1.3 billion of this growth occurring in Africa.^75^ Predictions also suggest that health risks will increase dramatically in Africa, with the endemic rate of zoonotic viruses more than tripling by 2070.^76^ Consequently, health services, particularly laboratory services, will have to support this demographic growth and high risk of emerging infectious diseases by providing services at low cost while maintaining quality.

Given the low scores recorded and the few numbers of accredited and BSL3 and BSL4 laboratories, it appears that the ECCAS countries have weak laboratory capacities in both human and veterinary medicine and must be strengthened. Building the laboratory workforce is another challenge that ECCAS countries face since laboratory work has long been down on the lists of priorities of most health ministries in the ECCAS region.

The Central Africa’s Regional Integrated Surveillance and Laboratory Network (RISLNET) meeting, which took place in Malabo in March 2019,^77^ revealed that only a few countries including Burundi, the Democratic Republic of Congo and Cameroon have their laboratory policies and strategic plans drawn up and validated. The Republic of Congo and São Tomé and Príncipe laboratory strategic plans remain to be validated. The Central African Republic, Chad, Gabon and Equatorial Guinea have no laboratory policies and no laboratory strategic plans. For those with laboratory policies and strategic plans, implementing them remain a challenge due to budgetary constraints.

#### Capacity strengthening through laboratory quality enhancement

**FIGURE 2:**
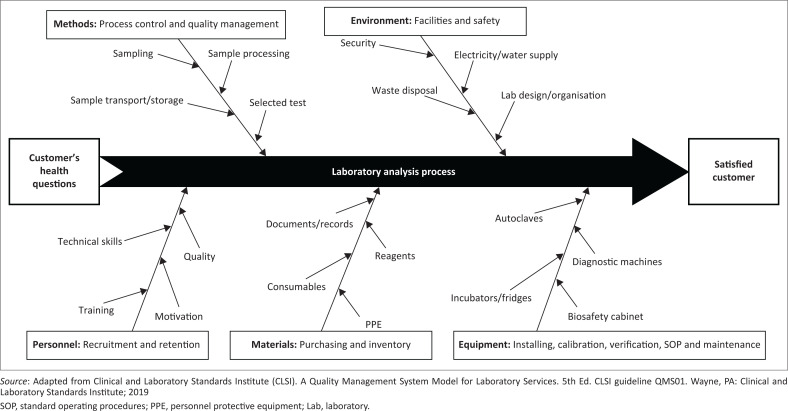
Fishbone diagram for medical laboratory analysis showing the focal elements in the capacity strengthening strategy.

The information provided by the laboratory must be accurate, timely and subjected to quality assurance procedures. In other words, laboratory results must be accurate. To this end, all aspects of laboratory activities must be reliable and the reporting of results must be correct to be used for clinical or public health purposes. The ISO divides the various laboratory analysis activities into three processes: pre-analytical, analytical, and post-analytical. Laboratory errors that may negatively impact patient management or public health policies occur at 32% – 75% in the pre-analytical phase, 13% – 32% in the analytical phase and 9% – 31% in the post-analytical phase.^78^ Therefore, as shown in Figure 2, capacity building strategies need to be designed to address all aspects of the laboratory analysis and organisation.^79^

The best way to strengthen capacity is to enrol laboratories within the WHO’s SLMTA process. This process allows a substantial improvement in the quality of laboratories even if they do not reach the end of the accreditation process.^11,12,13,80^ Unfortunately, few countries in the ECCAS zone have engaged their laboratories in the SLMTA process. And even for those countries that have signed up, the number of both public and private laboratories involved in the process is very low. For example, between 2012 and 2019, only 16 public and private laboratories in Cameroon were enrolled in SLMTA, but this country has 3279 public laboratories.^81^ Also, only 1.34% of the 1113 public and private laboratories in Burundi were engaged in the SLMTA process.^81^ To avoid a decline in performance, SLMTA-enrolled laboratories must continue follow-up performance and apply the lessons learned during the process and, most importantly, attract national political commitment.^82^

#### Moving towards integrated laboratory systems and networks

The Maputo declaration called for the development of national laboratory policies and national strategic laboratory plans. The call prioritises laboratory systems in the national health development plan.^2^ An integrated laboratory network can provide all primary diagnostic services needed for the care and treatment of patients without requiring them to go to different laboratories for specific tests.^83^ In resource-limited settings, such as in some African countries, the WHO recommends four operational levels of laboratories to better provide services in a national laboratory network^84^: Level I or Primary (health post and health centre laboratories that primarily serve outpatients), Level II or District level (laboratories in intermediate referral facilities), Level III or Regional or Provincial level (laboratories in a regional or provincial referral hospital that may be part of a regional or provincial health bureau), and Level IV or National or Multi-country Reference Laboratory (reference laboratory for one or more countries).

Thus, laboratory levels are determined by their diagnostic platforms as well as their functions. The national reference level carries out the most complex tests. A tiered, integrated laboratory network should meet the following criteria^83^: (1) provide quality-assured basic laboratory testing, (2) collect the common specimens, report results timeously, and use diagnostic platforms to detect different diseases within the same facility, and (3) increase capacity for introducing and using new and more complex technologies. An integrated laboratory should have the capacity to adequately monitor people with HIV/AIDS for tuberculosis, malaria or other opportunistic infections. It should also provide rapid molecular tests for multidrug-resistant tuberculosis in patients co-infected with HIV and tuberculosis to improve infection control and treatment outcomes. Therefore, the integrated network avoids wastage of already limited resources and the referral of patients outside the network for certain laboratory tests.^83^ In 1993, the WHO AFRO established an integrated laboratory network in 15 African countries, including Cameroon, the Central African Republic and the Democratic Republic of Congo, to support the Global Polio Eradication Initiative.^15^ The polio laboratories of these three ECCAS countries have also integrated measles, yellow fever, and rotavirus programmes.

More recently, the Africa Centre for Disease Control and Prevention established RISLNET within defined geographic regions of Africa including central Africa.^77^ The RISLNET aims to effectively support prevention, rapid detection, and response to current and emerging public health threats. The RISLNET operates under the One Health concept, integrating human and animal health laboratories and surveillance assets. The materialisation of the One Health concept is critical to efficiently prevent and respond to public health threats. As 65% of the recent major epidemics in the world have a zoonotic origin,^85^ there should be no division between disciplines in the human and animal health sectors. The One Health approach will enable the early identification of emergent zoonosis. This can be achieved through the simultaneous surveillance of both human and animal disease in integrated surveillance and laboratory systems or networks. The One Health approach, thus, mutualises resources and cuts costs. This was illustrated in Uganda and Nigeria, where during avian influenza outbreaks, HIV/AIDS diagnostic laboratories provided diagnostic support for avian influenza cases.^83^ In the fight against emerging AMR threats, a stronger laboratory system will allow the detection of resistance and provide data for better trend tracking and infection control. Also, standardised isolates banks, which would result from such a system, would support the research for better diagnostics and treatment.

### Limitations

In response to the coronavirus disease 2019 pandemic, several countries must have increased their response capacity by improving their laboratory capacity and biosafety levels. The laboratory capacities reported here may not have considered all the newly acquired capacities. This study was based on data available online; thus, a country’s laboratory capacity may not necessarily have been the subject of a study published on the Internet. Within the framework of the Regional Disease Surveillance Systems Enhancement project (REDISSE IV) currently underway in the ECCAS countries, an inventory of laboratory capacities is being conducted and will provide exhaustive data on laboratory capacities.

### Conclusion

Laboratory services are an essential component of a health system. In Africa, particularly in the ECCAS sub-region, the need for reliable laboratory systems is greatest due to the higher risk of VHF. Unfortunately, from this review, it is evident that laboratory capacity for disease and AMR surveillance and response is weak. Indeed, the capacities of laboratories in ECCAS countries are weak given the WHO’s JEE scores and the very limited number of high biosafety levels (BSL3 and BSL4) and accredited laboratories. There is, therefore, a pressing need to strengthen the laboratory capacities in the sub-region to cope with the risk of disease emergence, which is predicted to triple in the coming decades.
