# Corrigendum: Microbiology laboratories involved in disease and antimicrobial resistance surveillance: Strengths and challenges of the central African states

**DOI:** 10.4102/ajlm.v12i1.1913

**Published:** 2023-05-09

**Authors:** Passoret Vounba, Severin Loul, Ludovic F. Tamadea, Joël F.D. Siawaya

**Affiliations:** 1 Economic Community of Central African States (ECCAS) Commission/Fourth phase of the Regional Disease Surveillance Systems Enhancement Project (REDISSE IV), Libreville, Gabon; 2 Department of Laboratory Services, CHU Mère-Enfant Fondation Jeanne EBORI, Libreville, Gabon; 3 Regional Integrated Surveillance and Laboratory Network (RISLNET) for Central Africa, Libreville, Gabon

In the published article, Vounba P, Loul S, Tamadea LF, Siawaya JFD. Microbiology laboratories involved in disease and antimicrobial resistance surveillance: Strengths and challenges of the central African states. Afr J Lab Med. 2022;11(1), a1570. https://doi.org/10.4102/ajlm.v11i1.1570, there is an omission in the Acknowledgements statement. The corrected Acknowledgements statement appears below.

**The original incorrect wording:**

We sincerely thank Dr Skander Hathroubi (from Humboldt University of Berlin, Germany) for his contribution by reading and correcting the first draft of the manuscript.

**The revised and updated wording:**

We sincerely thank Dr Skander Hathroubi (from Humboldt University of Berlin, Germany) for his contribution by reading and correcting the first draft of the manuscript. This study was commissioned and supported by the Regional Disease Surveillance Systems Enhancement (REDISSE) project in Central Africa, which is financially supported by the World Bank and managed by the Economic Community of Central African States (ECCAS). We would like to thank ECCAS and the World Bank for supporting our efforts through REDISSE.

The authors apologise for this error. The correction does not change the study’s findings of significance or overall interpretation of the study’s results or the scientific conclusions of the article in any way.

